# The lncRNA NEAT1 promotes the epithelial-mesenchymal transition and metastasis of osteosarcoma cells by sponging miR-483 to upregulate STAT3 expression

**DOI:** 10.1186/s12935-021-01780-8

**Published:** 2021-02-05

**Authors:** Yan Chen, Jun Li, Jia-Kun Xiao, Lei Xiao, Bin-Wu Xu, Chen Li

**Affiliations:** 1 Department of Nephrology, The Second Affiliated Hospital of Nanchang University, Nanchang, 330006 People’s Republic of China; 2 Department of Orthopedic Surgery, The Second Affiliated Hospital of Nanchang University, No. 1 Minde Road, Nanchang, 330006 Jiangxi People’s Republic of China

**Keywords:** Osteosarcoma, miR-483, NEAT1, STAT3, Epithelial-mesenchymal transition (EMT), Metastasis

## Abstract

**Background:**

Osteosarcoma is one of the most prevalent primary bone tumours in adolescents. Accumulating evidence shows that aberrant expression of the long non-coding RNA (lncRNA) NEAT1 and microRNA-483 (miR-483) contribute to the epithelial-mesenchymal transition (EMT), invasion and metastasis of tumour cells. However, the potential regulatory effects of NEAT1 and miR-483 on the EMT of osteosarcoma remain elusive.

**Methods:**

The expression of the NEAT1, miR-483, signal transducer and activator of transcription-1 (STAT1), STAT3, and EMT-associated markers was measured using qRT-PCR or western blotting. NEAT1 overexpression or knockdown was induced by lentivirus-mediated transfection. A luciferase reporter assay was employed to confirm the association between NEAT1/miR-483 and miR-483/STAT3. RNA immunoprecipitation (RIP) was also performed to verify the NEAT1 and miR-483 interaction. Wound healing and transwell assays were implemented to assess the migration and invasion of U2OS cells. Unilateral subcutaneous injection of U2OS into nude mice was performed to investigate tumour metastasis in vivo.

**Results:**

The expression of miR-483 was downregulated in both osteosarcoma cell lines and osteosarcoma tissues. The overexpression of miR-483 suppressed the migration, invasion, and expression of EMT-associated proteins in U2OS cells, while simultaneous overexpression of STAT3 partially relieved this suppression. Mechanistically, miR-483 specifically targeted the 3′ untranslated region (3′UTR) of STAT3 and repressed its expression. However, NEAT1 sponged miR-438, increased STAT3 expression, and repressed STAT1 expression, subsequently increasing the EMT of osteosarcoma cells. The knockdown of NEAT1 in transplanted U2OS cells impaired the liver and lung metastases of osteosarcoma in nude mice. Moreover, NEAT1 silencing inhibited the mesenchymal- epithelial transition (MET) of osteosarcoma at metastasis sites.

**Conclusions:**

The lncRNA NEAT1/miR-483/STAT3 axis plays a crucial role in regulating the metastasis of osteosarcoma and potentially represents one appealing therapeutic target for osteosarcoma treatment in the future.

## Background

Osteosarcoma is one of the most common primary bone malignancies worldwide, and it typically occurs in children and young adults aged > 10 years [1]. From 2011 to 2015, the percentage of osteosarcoma cases among all childhood cancers in children aged from birth to 14 years was 2%, and the 5-year survival rate was only 69.6%; for youths aged from 15 to 19 years, the incidence was 3% and the 5-year survival rate decreased to 65.7% [2]. Osteosarcoma cells originate from mesenchymal tissues and have strong proliferative, invasive and angiogenetic abilities, facilitating their early metastasis with a high degree of malignancy [3]. Moreover, according to recent statistics, the 5-year survival rate is approximately 77% for patients with localized osteosarcoma and only 27% for patients with metastasized osteosarcoma at diagnosis (data from the website of the American Cancer Society). Currently, the pathogenesis of osteosarcoma remains elusive, and common treatment approaches include surgery, chemotherapy and radiotherapy. However, an effective targeted therapy for osteosarcoma is still lacking in clinical practice. Thus, additional basic and clinical studies on osteosarcoma are required in cancer research.

Signal transducer and activator of transcription-3 (STAT3) is a member of the STAT family of transcription factors, which is primarily phosphorylated and activated by cytokine receptor-associated Janus kinases (JAKs) [4]. Subsequently, dimerized STAT3 translocates into the cell nucleus and promotes the transcription of some anti-apoptotic and pro-proliferative genes [5]. However, dysregulation of STAT3 is closely related to the survival, proliferation, and metastasis of cancer cells [6]. For example, Tu and colleagues found that IL-6 secreted from mesenchymal stem cells (MSCs) activated STAT3 in osteosarcoma cells and promoted their survival and metastasis [7]. Studies by the Heiko Hermeking group and Junjeong Choi group further confirmed that IL-6/STAT3 signalling facilitates the epithelial-mesenchymal transition (EMT) and metastasis of colorectal cancer cells (CRC) [8] and breast cancer cells [9], respectively. E-cadherin, N-cadherin, Vimentin and Snail are major proteins involved in the EMT [10]. During the EMT of osteosarcoma, E-cadherin expression is repressed [11], whereas the expression of N-cadherin, Vimentin and Snail is significantly upregulated [12–14]. As shown in the study by Xiong et al., STAT3 overexpression significantly reduces E-cadherin expression and increases N-cadherin and Vimentin expression in CRC cells to subsequently increase the invasion of CRC and resistance to apoptosis [15]. Thus, treatments targeting STAT3 may represent one promising approach to block the EMT and metastasis of osteosarcoma cells.

MicroRNAs (miRNAs) are a family of small non-coding RNAs that modulate gene expression at the post-transcriptional level [16]. Based on emerging evidence, miRNAs are also implicated in the initiation and progression of osteosarcoma [17]. As one oncogenic molecule, miR-483 is upregulated in most tumours [18]. For example, Arrighetti et al. found that miR-483 overexpression in ovarian cancer interferes with the proliferation of these tumour cells by targeting PRKCA (encoding PKC-α) and subsequently protects them from platinum-induced DNA damage [19]. According to Che et al., miR-483 promotes proliferation, migration, and invasion and induces chemoresistance in Wilms tumour cells [20]. However, in some tumour types, such as glioma, osteosarcoma, breast cancer and angiosarcoma, miR-483 is downregulated, indicating that it also functioned as a tumour suppressor [21–23]. In our lab, we recently identified STAT3 as a potential target of miR-483 through bioinformatics predictions. Thus, we speculated that miR-483 might participate in the regulation of osteosarcoma through STAT3.

Long non-coding RNAs (lncRNA) are a class of RNA with a length of more than 200 nucleotides that are expressed in eukaryotic cells and play crucial roles in various biological activities [24]. Nuclear paraspeckle assembly transcript 1 (NEAT1) is one lncRNA that has been discovered in recent years and is implicated in the progression of various tumours. For example, Chen et al. reported positive correlations between NEAT1 expression and the tumour grade and metastasis of ovarian cancer and the poor prognosis of patients with ovarian cancer [22]. Sun et al. and Zhang et al. independently revealed the significant upregulation of NEAT1 expression in patients with non-small cell lung cancer (NSCLC) and breast cancer, respectively, which promoted tumour cell growth and metastasis through distinct mechanisms [25, 26]. As shown in a recent study by Zhang and colleagues, NEAT1 promotes the proliferation, invasion and migration of osteosarcoma cells via the miR-339-5p/TGF-β1 pathway, which subsequently facilitated the progression of osteosarcoma [27]. However, the role of NEAT1 in the EMT of osteosarcoma has not been reported. Recently, our lab identified putative binding sites for miR-483 in NEAT1 using a bioinformatics analysis, suggesting that NEAT1 might participate in the mechanism regulating the EMT and metastasis of osteosarcoma by sponging miR-483.

In the present study, miR-483 inhibition promoted the EMT of osteosarcoma cells. Based on the results of the mechanistic study, miR-483 targeted STAT3 and restrained its expression, while NEAT1 sponged miR-483 to restore STAT3 function in the EMT. Moreover, the effects of NEAT1 on promoting the EMT and metastasis of osteosarcoma cells were confirmed in vivo. Overall, the identification of NEAT1/miR-483/STAT3 axis contributed to the elucidation of the regulatory network involved in the progression of osteosarcoma and provided insights into the prognosis and development of targeted therapy for osteosarcoma in the future.

## Methods

### Clinical studies

Human osteosarcoma tumour tissues and normal tissues were obtained from 20 patients via surgical excision, which was approved by the Ethics Committee of the Second Affiliated Hospital of Nanchang University (Approval number: Review [2018] No. (080). Nanchang, Jiangxi, China). Written informed consent forms were signed by all enrolled patients. All samples were immediately frozen in liquid nitrogen and stored at − 80 °C until RNA extraction and qRT-PCR analysis.

### Cell culture and treatment

The human osteosarcoma cell lines U2OS, MG-63 and Saos-2, and the human normal osteoblast cell line hFOB 1.19 were purchased from the American Type Cell Culture (ATCC, Manassas, VA, USA) and were certificated by STR genotyping. U2OS cells and Saos-2 cells were cultured in McCoy’s 5a medium (Gibco, Grand Island, NY, USA) supplemented with 10% foetal bovine serum (FBS, Gibco, Grand Island, NY, USA) and 1% penicillin and streptomycin (Gibco, Grand Island, NY, USA). MG-63 cells were grown in Dulbecco’s Modified Eagle’s Medium (DMEM, Gibco, Grand Island, NY, USA) supplemented with 10% FBS (Gibco, Grand Island, NY, USA) and 1% penicillin and streptomycin (Gibco, Grand Island, NY, USA). hFOB 1.19 cells were cultured in a 1:1 mixture of Ham’s F12 Medium and DMEM, which was supplemented with 2.5 mM l-glutamine, 0.3 mg/mL G418, 10% FBS, and 1% penicillin and streptomycin (Gibco, Grand Island, NY, USA). hFOB 1.19 cells were maintained in a humidified incubator at 34 °C with 5% CO_2_, and other cells were maintained at 37 °C with 5% CO_2_. All the cells were tested for mycoplasma contamination monthly. Mycoplasma-free cells were used in subsequent experiments. When cells reached approximately 80% confluence, they were passaged by trypsinization. The detached cells were seeded into a new flask at a density of 2.5 × 10^3^ cells/cm^2^. All the osteosarcoma cell lines used for in vitro and in vivo experiments were maintained in logarithmic growth phase. U2OS cells were treated with 15 µM Stattic (Sigma-Aldrich, USA) for 24 h to inhibit STAT3 activity, and DMSO was used as the vehicle control.

### Plasmid construction, transfection, and target cell infection

Exogenous gene overexpression and knockdown were performed using stable lentivirus transfection or Lipofectamine 3000-mediated transient transfection, respectively.

The human NEAT1 and STAT3 genes were synthesized by Sangon Biotech (Shanghai, China), subcloned into the multiple cloning site of the pLenti6.3/V5-DEST expression plasmid, and then transfected into 293T cells along with the pLP1 and pLP2 packaging plasmids and pLP/VSVG envelop plasmid using Lipofectamine 3000 (Invitrogen, USA) to produce NEAT1- and STAT3-overexpressing lentiviruses for the stable overexpression of NEAT1 and STAT3 in U2OS cells. The titre of the lentivirus was determined, and then U2OS and MG-63 cells were infected with the appropriate lentivirus to generate stable NEAT1- or STAT3-overexpressing cells.

An shRNA targeting NEAT1 and negative control shRNA were constructed in the pLKO.1 vector with the sequences listed in Table 1, which were synthesized by Sangon Biotech, to stably knock down NEAT1 in U2OS cells. The pLKO.1-shNEAT1 or pLKO.1-shNC plasmid was then transfected into 293T cells together with psPAX2 and pMD2.G plasmids using Lipofectamine 3000 to produce an shRNA-containing lentivirus. The titre of the lentivirus was determined and it was transduced into U2OS cells to obtain stable NEAT1 knock down (KD) cells.

**Table 1:** shRNA sequences used in plasmid construction

shRNA name	Sequence (5′-3′)
shNEAT1	CCGGTGGCTAGCTCAGGGCTTCAGCTCGAGCTGAAGCCCTGAGCTAGCCATTTTTG
shNC	CCGGCCTAAGGTTAAGTCGCCCTCGCTCGAGCGAGGGCGACTTAACCTTAGGTTTTTG

The miR-483 mimics, miR-483 inhibitor or corresponding negative control (NC) miRNA were synthesized by Sangon Biotech and transfected into U2OS and MG-63 cells using Lipofectamine 3000 according to the manufacturer’s instructions to transiently knock down or overexpress miR-483. Briefly, 9 μL of Lipofectamine 3000 were diluted in 150 μL of Opti-MEM. Then, 50 nM of miR-483 mimics, 50 nM of miR-483 inhibitor, or 50 nM of the NC miRNA was added to the diluted Lipofectamine 3000 reagent and mixed well. After an incubation at 22 °C for 5 min, the miRNA-lipid complex was evenly added to the U2OS or MG-63 cells in a 6-well plate. 24 h after transfection, the U2OS and MG-63 cells were harvested for subsequent experiments.

### Quantitative reverse transcription-polymerase chain reaction (qRT-PCR)

Total RNA were isolated from tissues or U2OS and MG-63 cells using TRIzol (Invitrogen, Carlsbad, CA, USA) according to the manufacturer’s instructions. The first-strand cDNAs were then obtained from 2 μg of total RNA with PrimeScript RT reagent Kit (for mRNAs, Takara, Dalian, China) or TaqMan MicroRNA Reverse Transcription Kit (for miR-483, Thermo Fisher Scientific, USA). Next, qRT-PCR was conducted using a 7900HT real-time PCR system (Thermo Fisher Scientific, Carlsbad, CA, USA) with SYBR Green qPCR Master Mix (Thermo Fisher Scientific, Carlsbad, CA, USA) and 50 ng of cDNAs as the template. The fold change in the expression of target genes was calculated using the 2^−△△Ct^ method, with GAPDH or U6 serving as the internal controls for mRNAs/lncRNAs and miRNAs, respectively. The primers used for qRT-PCR are listed in Table 2.

**Table 2:** The primers used in quantitative real-time PCR

Primer name	Sequence (5′-3′)
Human NEAT1 forward	GUCUGUGUGGAAGGAGGAATT
Human NEAT1 reverse	UUCCUCCUUCCACACAGACTT
Human miR-483 forward	GTCGTATCCAGTGCAGGGTCCGAGGTATTCGCACTGGATACGACAAGACG
Human miR-483 reverse	GGCTCACTCCTCTCCTCC
Human STAT3 forward	CAGCAGCTTGACACACGGTA
Human STAT3 reverse	AAACACCAAAGTGGCATGTGA
Mouse NEAT1 forward	GGGGCCACATTAATCACAAC
Mouse NEAT1 reverse	CAGGGTGTCCTCCACCTTTA
Mouse miR-483 forward	GTCGTATCCAGTGCGTGTCGTGGAGTCGGCAATTGCACTGGATACGACAAGACGG
Mouse miR-483 reverse	AATTTCACTCCTCCCCTCC
Mouse STAT3 forward	TGGTGTCCAGTTTACCACGA
Mouse STAT3 reverse	CCCACATCTCTGCTCCCTAA
Human E-cadherin forward	CGAGAGCTACACGTTCACGG
Human E-cadherin reverse	GGGTGTCGAGGGAAAAATAGG
Human N-cadherin forward	TTTGATGGAGGTCTCCTAACACC
Human N-cadherin reverse	ACGTTTAACACGTTGGAAATGTG
Human Vimentin forward	GACGCCATCAACACCGAGTT
Human Vimentin reverse	CTTTGTCGTTGGTTAGCTGGT
Human Snail forward	TCGGAAGCCTAACTACAGCGA
Human Snail reverse	AGATGAGCATTGGCAGCGAG
Human GAPDH forward	GGACACAATGGATTGCAAGG
Human GAPDH reverse	TAACCACTGCTCCACTCTGG
Human U6 forward	CTCGCTTCGGCAGCACA
Human U6 reverse	AACGCTTCACGAATTTGCGT
Mouse GAPDH forward	AGCCCAAGATGCCCTTCAGT
Mouse GAPDH reverse	AGCCCAAGATGCCCTTCAGT
Mouse U6 forward	GCTTCGGCAGCACATATACTAAAAT
Mouse U6 reverse	CGCTTCACGAATTTGCGTGTCAT

### Western blotting

U2OS and MG-63 cells or tumour tissues were directly lysed or homogenized in RIPA buffer (Sangon Biotech, Shanghai, China) supplemented with a 1 × protease inhibitor cocktail. Afterwards, the total protein concentration in lysates was measured using a BCA Kit (Tiangen Biotech, Beijing, China). 30 μg of denatured proteins were then loaded in 10% SDS-PAGE gels, electrophoretically separated, and transferred to PVDF membranes (Millipore Corp, Bedford, MA). Next, the membrane was blocked with 10% BSA for 1 h and then incubated with the indicated primary antibodies at 4 °C overnight: anti-E-cadherin (1:1000, Cat No: 3195, Cell Signaling Technology (CST), USA), anti-N-cadherin (1:1000, Cat No: 13116, CST, USA), anti-Vimentin (1:1000, Cat No: 5741, CST, USA), anti-Snail (1:2000, Cat No: 3879, CST, USA), anti-STAT1 (1:1000, Cat No: 14994, CST, USA), anti-STAT3 (1:2000, Cat No: 12640, CST, USA), anti-phospho-STAT3 (Tyr705) (1:2000, Cat No: 9145, CST, USA), and anti-GAPDH (1:5000, Cat No: 2118, CST, USA). After 3 washes with Tris-buffered saline containing 0.1% Tween 20 (TBST), the PVDF membrane was then incubated with a goat anti-rabbit IgG-HRP antibody (1:5000, Cat No: 7074, CST, USA) for 1 h. The PVDF membranes were immersed in ECL substrate (Piece, Thermo Fisher Scientific, USA) for chemiluminescence signal development, and the bands were exposed to autoradiography films in a dark room. Band intensities were then quantified using ImageJ software (NIH, Bethesda, MD, USA).

### Wound healing assay

Wound healing assays were performed using the method described in a previous study [28]. U2OS cells were seeded in a 12-well plate at a density of 5.0 × 10^5^ cells/well. 24 h later, when the cells reached approximately 80% confluence, a sterile 200 μL pipette tip was used to create a straight-line scratch in the cell monolayer across the centre of the wells, and the detached cells were then gently removed with culture medium. Afterwards, the culture medium was replaced with serum-free McCoy’s 5a medium and the gap in the monolayer was imaged using an inverted microscope (Olympus IX71, Tokyo, Japan). The scratched U2OS cells were then cultured for another 24 h in serum-free McCoy’s 5a medium, washed with PBS and fixed with 4% paraformaldehyde (PFA). The gap in the monolayer was imaged again using the same inverted microscope. Five randomly selected images were captured for each gap in one well, which were then analysed using ImageJ software (NIH, Bethesda, MD, USA).

### Transwell migration and invasion assays

The methods for the transwell migration and invasion assays have been reported in a previous study [29]. For the migration assay, the upper transwell insert (8-μm pore size; Corning Costar, Cambridge, MA, USA) was directly placed in one well of a 24-well plate. For the invasion assay, 50 μL of Matrigel gel (BD Bioscience, USA) were evenly spread on the upper surface of the transwell insert (8-μm pore size; Corning Costar, Cambridge, MA, USA) that was then gently placed in a well of 24-well plate and incubated overnight to allow the gel to solidify. After trypsinization, the U2OS cells were re-suspended in serum-free McCoy’s 5a medium and the cell density was adjusted to 2 × 10^5^ cells/mL. Afterwards, 250 μL of McCoy’s 5a medium supplemented with 10% FBS were added to the bottom chamber of the 24-well plate, 100 μL of the U2OS cell suspension in serum-free McCoy’s 5a medium were added to the upper chamber, and then the plate was placed in the incubator for 24 h. After that, the inserts were collected, the non-invading or non-migrating cells on the upper surface were removed with a cotton swab, cells invaded or migrated to the bottom well were directly fixed with 4% PFA for 15 min and subsequently stained with crystal violet for 15 min, which was captured on a fluorescence microscope (IX-71, Olympus, Japan). The number of cells on the bottom well was quantified by Image J software (NIH, Bethesda, MD, USA).

### RNA immunoprecipitation

RIP was performed as described in a previous study [30] with a RIP RNA-binding protein immunoprecipitation Kit (Millipore, USA) according to the manufacturer’s instructions to confirm the interaction between miR-483 and NEAT1 in U2OS and MG-63 cells. Briefly, U2OS and MG-63 cells were initially lysed in RIP lysis buffer and then incubated with magnetic beads conjugated with a rabbit anti-Argonaute2 (Ago2) antibody (Cat No: 2897, CST, USA) or rabbit IgG isotype control (Cat No: 3900, CST, USA) in RIP immunoprecipitation buffer at 4 °C overnight. The beads were collected with a magnetic separator, washed with RIP wash buffer, and then treated with Proteinase K at 55 °C for 30 min. Next, the supernatant was collected and the RNAs were finally extracted with phenol–chloroform before analysis using qRT-PCR.

### Luciferase reporter activity assay

The binding sites for miR-483 in the STAT3 and lncRNA NEAT1 sequences were predicted with the Starbase database (http://starbase.sysu.edu.cn/index.php). The wild type or mutated 3′UTR of the lncRNA NEAT1 or STAT3 (NEAT1 or STAT3-WT or NEAT1 or STAT3-MUT) were sub-cloned into the pmiR-RB-Report™ vector, which was synthesized by Ribobio company (Guanzhou, China), to verify these interactions. Afterwards, the luciferase constructs and miR-483 mimics or mimics NC were transfected into U2OS and MG-63 cells with Lipofectamine 3000 (Invitrogen, USA). 48 h later, the U2OS and MG-63 cells were harvested, lysed and luciferase activity was measured with the Dual Luciferase Assay Kit (Promega, US) according to the manufacturer’s instructions. Both Renilla and firefly luciferase activities were measured using a microplate spectrophotometer (NEO, Bio-Tek, USA). The Renilla luciferase activity was used as an internal reference.

### Nude mouse model

Female BALB/C nude mice were obtained from Shanghai SLAC Laboratory Animal Co., Ltd. (Shanghai, China) at the age of 3–4 weeks. Upon arrival, the animals were maintained in pathogen-free animal facilities at the Second Affiliated Hospital of Nanchang University (Nanchang, Jiangxi, China). All experimental protocols described in this study were reviewed and approved by the Animal Ethics Committee (AEC) of the Second Affiliated Hospital of Nanchang University (Approval number: Review [2018] No. (017). Nanchang, Jiangxi, China). The maximum tumour volumes were in compliance with the guidelines of the AEC. Nude mice were randomly allocated to three groups with 5 mice per group to establish liver and lung metastases of osteosarcoma. One group was the negative control without any treatment, and the other two groups were subcutaneously injected with 5 × 10^6^ U2OS cells that were transfected with the control shRNA plasmid (sh-NC) or NEAT1 KD plasmid (sh-NEAT1) into the left outer flank. The tumour volume was measured with callipers every 7 days for 6 weeks. Six weeks after the injection, the mice were euthanized, and the liver and lung were surgically dissected to compare the number and volume of metastatic tumours. The primary (injection site) and metastatic (liver and lung) tumour tissues were then collected. Normal lung and liver tissues were collected from mice in the negative control group as the control samples. Images of primary and metastatic tumour tissues were first captured, and the tissues were weighed and subjected to qRT-PCR, western blotting and haematoxylin & eosin (HE) staining.

### HE staining

Mouse lung and liver tissues were initially divided into small pieces with a size of approximately 2-3 mm^3^ and then fixed with 4% PFA overnight. Afterwards, these tissues were embedded in paraffin and sectioned at a thickness of 5 μm. The sections were then dehydrated with xylol and different concentrations of ethanol, followed by brief washes and staining of the cell nuclei with a haematoxylin solution for 10 min. After rinses, the stained sections were differentiated in 0.3% HCl-ethanol for 30 s. Next, they were washed with running water for 1 min, the blue colour was developed in PBST for 1 min, washed with running water for another 1 min and rinsed with 95% ethanol for 10 s. Samples were then counterstained with an eosin staining solution for 2 min. After washes and dehydration, HE-stained sections were visualized and photographed using a fluorescence microscope (IX-51, Olympus, Japan). Images of five randomly selected areas were captured for each sample.

### Statistical analysis

All experiments reported in this study were performed at least three times, which referred to biological replicates. Each biological replicate consisted of 3 wells containing cells of the same passage, which referred to technical replicates. Data are presented as the mean ± the standard deviation (SD), and data were analysed using SPSS 19.0 software. Unpaired two-tailed Student’s *t*-test was applied to compare the difference between two groups. One-way analysis of variance (ANOVA) followed by Tukey’s post hoc test was used for comparisons among multiple groups. Differences were considered significant when the P value was less than 0.05.

## Results

### miR-483 inhibits the EMT in osteosarcoma cells

**Fig. 1:**
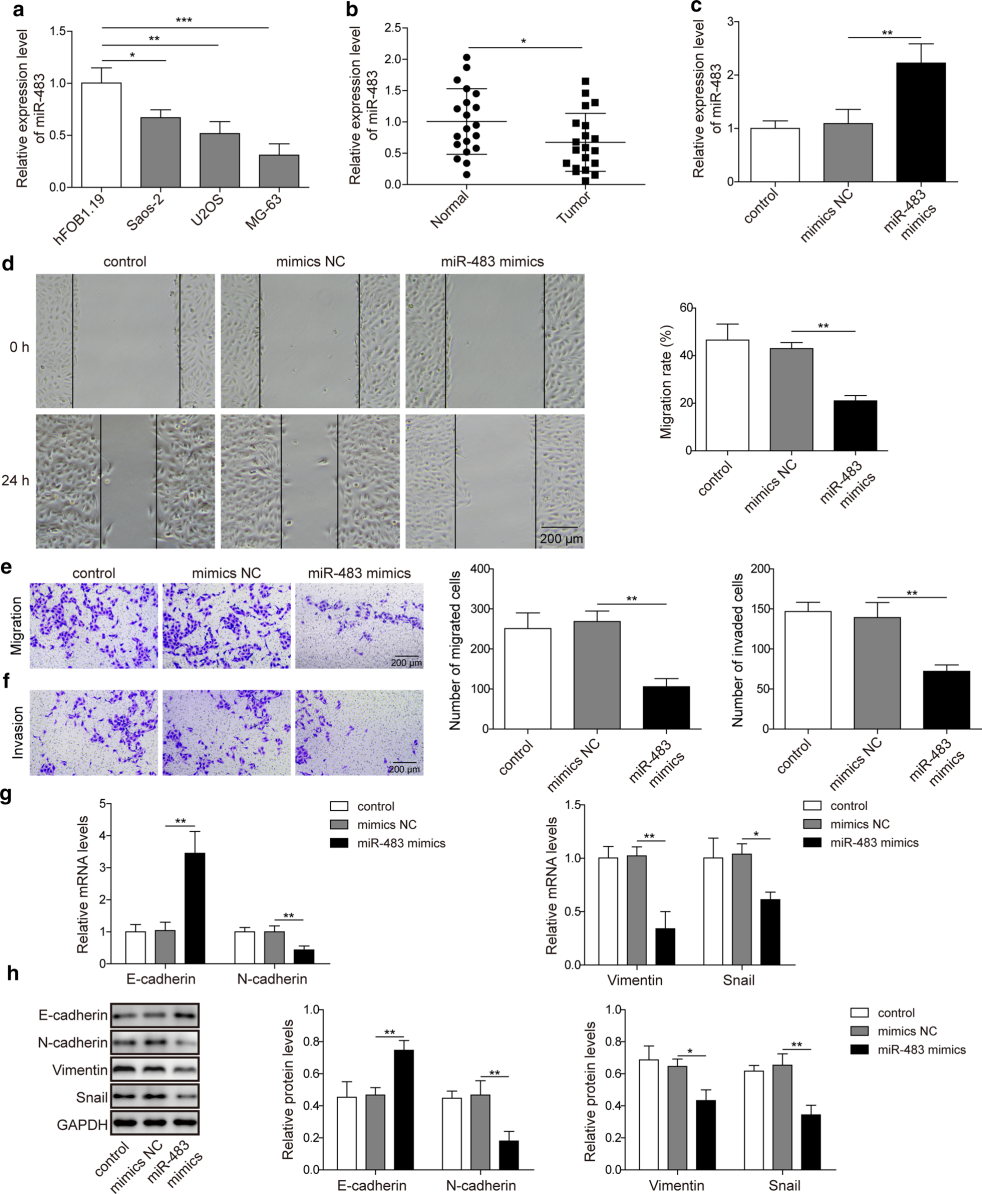
miR-483 inhibits the EMT in osteosarcoma cells. **a** The relative expression of miR-483 in the osteosarcoma cell lines Saos-2, U2OS, and MG-63 was measured using RT-qPCR. The human normal osteoblast cell line hFOB.19 was the control cell line. The expression of miR-483 was normalized to U6. **b** The relative expression of miR-483 in normal tissues and osteosarcoma tissues excised from patients was assessed using RT-qPCR (n = 20). The expression of miR-483 was normalized to U6. **c**–**h** U2OS cells were un-transfected or transfected with a negative control miRNA (NC) or miR-483 mimics. **c** The relative expression of miR-483 in U2OS cells was measured using qRT-PCR and normalized to U6 expression. **d**–**e** The migration of U2OS cells was measured using a scratch wound healing assay (**d**) or transwell migration assay (**e**). Scale bar: 200 μm. **f** The invasion of U2OS was assessed using the transwell invasion assay. Scale bar: 200 μm. **g** The relative expressions of N-cadherin, E-cadherin, Vimentin, and Snail in U2OS cells were determined using qRT-PCR. The mRNA levels were normalized to the GAPDH mRNA. **h** The protein levels of N-cadherin, E-cadherin, Vimentin, and Snail in U2OS cells were examined using western blotting. GAPDH served as the input control. All experiments were performed for least three times in this study, which referred to biological replicates. Each biological replicate was conducted in 3 wells with cells of the same passage, which referred to technical replicates. Error bars denoted mean ± SD. *p* values were calculated using paired Student’s *t*-test (B) or one-way analysis of variance (ANOVA) followed by Tukey’s post hoc test (A, C-H). ***p < 0.001, **p < 0.01 and *p < 0.05

We initially determined the expression of miR-483 in several common osteosarcoma cell lines and normal osteoblast cells, osteosarcoma tissues and normal tissues using qRT-PCR. The expression of miR-483 was downregulated in both osteosarcoma cell lines (Fig. 1a; p < 0.05, p < 0.01 and p < 0.001 for Saos-2, U2OS, and MG-63 cells, respectively) and osteosarcoma tissues (Fig. 1b; p < 0.05) compared normal osteoblast cells and normal tissues. Since U2OS cell had the moderate expression of miR-483 compared to Saos-2 and MG-63 cells, and it was one commonly used osteosarcoma cell line in extensive studies, we thus selected U2OS cells to study the effects of miR-483 on the EMT of osteosarcoma cells. U2OS cells were transfected with miR-483 mimics or mimics NC to overexpress miR-483 expression. The qRT-PCR results confirmed that miR-483 expression was evidently increased in miR-483 mimics-transfected cells, but not in mimics NC-transfected cells or un-transfected cells (Fig. 1c; p < 0.01). We then explored the effect of miR-483 on the migration and invasion of U2OS cells using wound healing and transwell assays, respectively. The wound healing data revealed that overexpression of miR-483 with miR-483 mimics substantially impaired the migration of U2OS cells (Fig. 1d; p < 0.01). Consistent with these findings, the transwell migration assay revealed miR-483 overexpression potently inhibited the migration of U2OS cells (Fig. 1e; p < 0.01). Moreover, miR-483 overexpression also attenuated the invasion of U2OS cells in the transwell invasion assay (Fig. 1f; p < 0.01). In addition, we assessed the expression of EMT-related proteins, such as E-cadherin, N-cadherin, Vimentin, and Snail, in cells transfected with miR-438 mimics. According to both the qRT-PCR and western blotting experiments, miR-483 overexpression reduced the expression of the mesenchymal markers N-cadherin and Vimentin and the EMT-related transcription factor Snail. However, miR-483 overexpression increased the expression of the epithelial marker E-cadherin (Fig. 1g,h; p < 0.01 for E-cadherin, N-cadherin, Vimentin, and p < 0.05 for Snail in qRT-PCR, respectively; p < 0.01 for E-cadherin, N-cadherin, Snail, and p < 0.05 for Vimentin in western blotting, respectively). Based on these findings, miR-483 inhibited the EMT of osteosarcoma cells.

### miR-483 targets STAT3 and reduces its expression

**Fig. 2:**
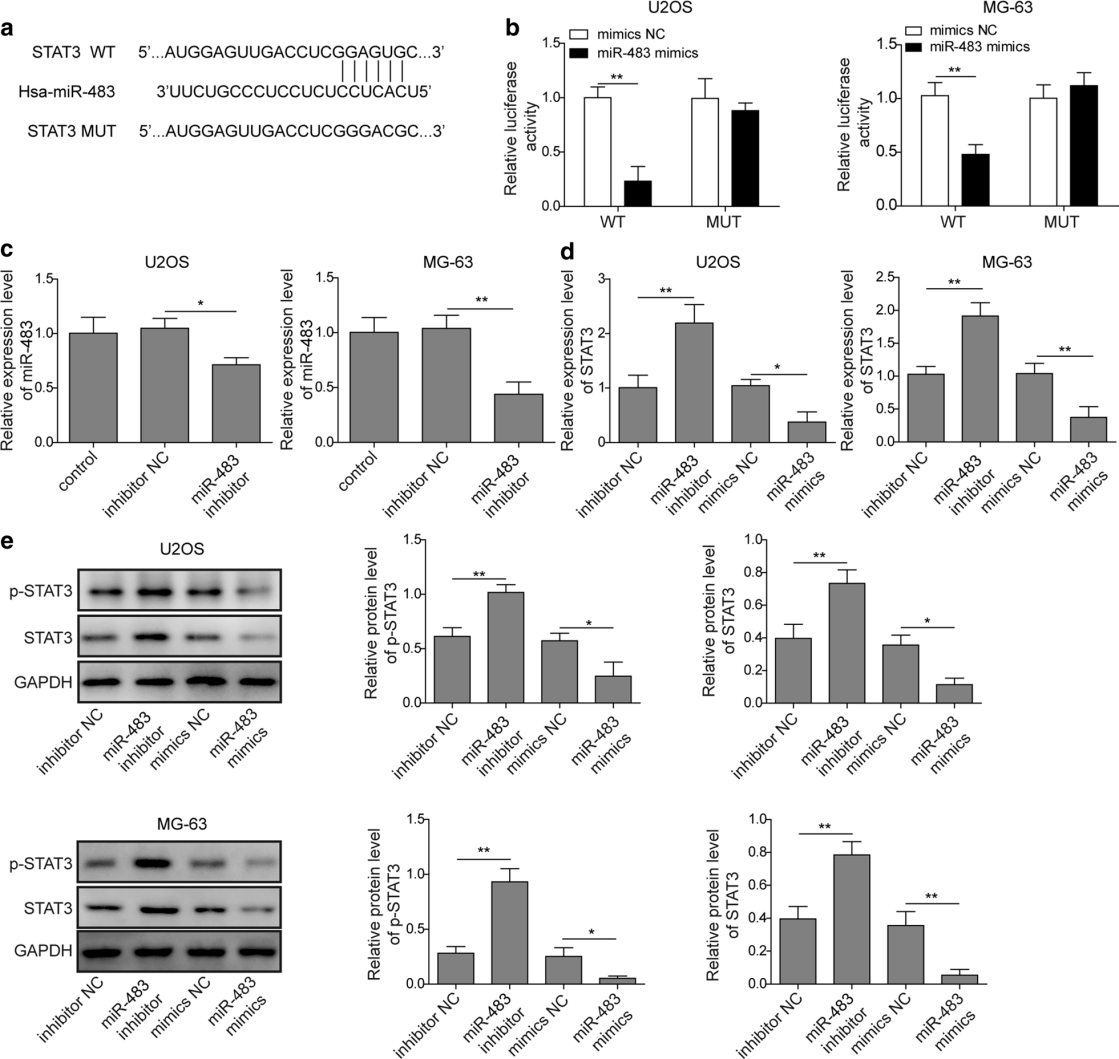
miR-483 targets STAT3 and reduces its expression. **a** The potential binding site for miR-483 in the 3′UTR of STAT3 according to the bioinformatics prediction from the Starbase database. **b** U2OS and MG-63 cells were transfected with a luciferase plasmid containing the wild type 3′UTR or mutant 3′UTR of STAT3 along with miR-483 mimics or mimics NC. The interaction between miR-483 and the 3′UTR of STAT3 was examined using a dual luciferase assay. **c**–**e** U2OS and MG-63 cells were un-transfected or transfected with inhibitor NC, miR-483 inhibitor, mimics NC, or miR-483 mimics. **c** The relative expression of miR-483 in control, inhibitor NC- or miR-483 inhibitor-transfected U2OS and MG-63 cells was measured using qRT-PCR. The expression of miR-483 was normalized to U6. **d** The relative expression of STAT3 in U2OS and MG-63 cells was also measured using qRT-PCR. **e** The levels and phosphorylation of STAT3 in U2OS and MG-63 cells were examined using western blotting. GAPDH served as the input control. All experiments reported in this study were performed at least three times, which referred to biological replicates. Each biological replicate consisted of 3 wells with cells of the same passage, which referred to technical replicates. Error bars denoted mean ± SD. *p* values were calculated using unpaired two-tailed student’s *t*-test (**b**) or one-way analysis of variance (ANOVA) followed by Tukey’s post hoc test (**c**–**e**). **p < 0.01 and *p < 0.05

As shown in numerous studies, miRNAs modulate the expression of target genes through two mechanisms: (1) binding to the target gene like an siRNA and degrading the target mRNA; and (2) binding to the 3′UTR of the target mRNA to inhibit translation. We screened the potential targets of miR-483 with bioinformatics methods using the Starbase database to ascertain the mechanism by which miR-483 suppresses the EMT of osteosarcoma cells. Unexpectedly, the 3′UTR of the STAT3 mRNA contained one putative binding site for miR-483 (Fig. 2a). To verify this prediction, we then conducted a luciferase reporter assay by co-transfecting luciferase constructs containing the firefly luciferase gene and the wild type or mutant 3′UTR of STAT3 along with miR-483 mimics or mimics NC into U2OS and MG-63 cells, respectively. The overexpressed miR-483 bound to the 3′UTR of the STAT3 mRNA, which inhibited the translation of fused luciferase mRNAs and reduced firefly luciferase activity. However, mutation of the predicted binding sites completely abrogated this interaction and restored the firefly luciferase activity in both U2OS and MG-63 cells (Fig. 2b; p < 0.01). Next, we quantified the levels of miR-483 and the STAT3 mRNA in U2OS and MG-63 cells transfected with miR-483 mimics, inhibitor, or corresponding negative controls (NCs) using qRT-PCR to further confirm the reciprocal regulation between miR-483 and STAT3. Transfection of the miR-483 inhibitor significantly reduced miR-483 expression in U2OS and MG-63 cells (Fig. 2c; p < 0.05 for U2OS cells; p < 0.01 for MG-63 cells). In contrast, miR-483 silencing increased STAT3 expression. However, transfection of the miR-483 mimics potently suppressed STAT3 expression (Fig. 2d; p < 0.01 for miR-483 inhibitor and p < 0.05 for miR-483 mimics in U2OS cells; p < 0.01 for miR-483 inhibitor and mimics in MG-63 cells). We further examined the level and phosphorylation of the STAT3 protein in U2OS and MG-63 cells exposed to the same treatments. The miR-483 mimics significantly reduced the levels and phosphorylation of the STAT3 protein, while the miR-483 inhibitor exerted the opposite effect (Fig. 2e; p < 0.01 for miR-483 inhibitor and p < 0.05 for miR-483 mimics for p-STAT3 and STAT3 in U2OS cells, respectively; p < 0.01 for miR-483 inhibitor and p < 0.05 for miR-483 mimics for p-STAT3 in MG-63 cells, respectively; p < 0.01 for miR-483 inhibitor and mimics for STAT3 in MG-63 cells). In summary, the consistent results in both U2OS and MG-63 cells indicated that miR-483 targeted the 3′UTR of STAT3 and negatively regulated its expression and phosphorylation.

### miR-483 inhibits the EMT in osteosarcoma cells by suppressing STAT3 expression

**Fig. 3:**
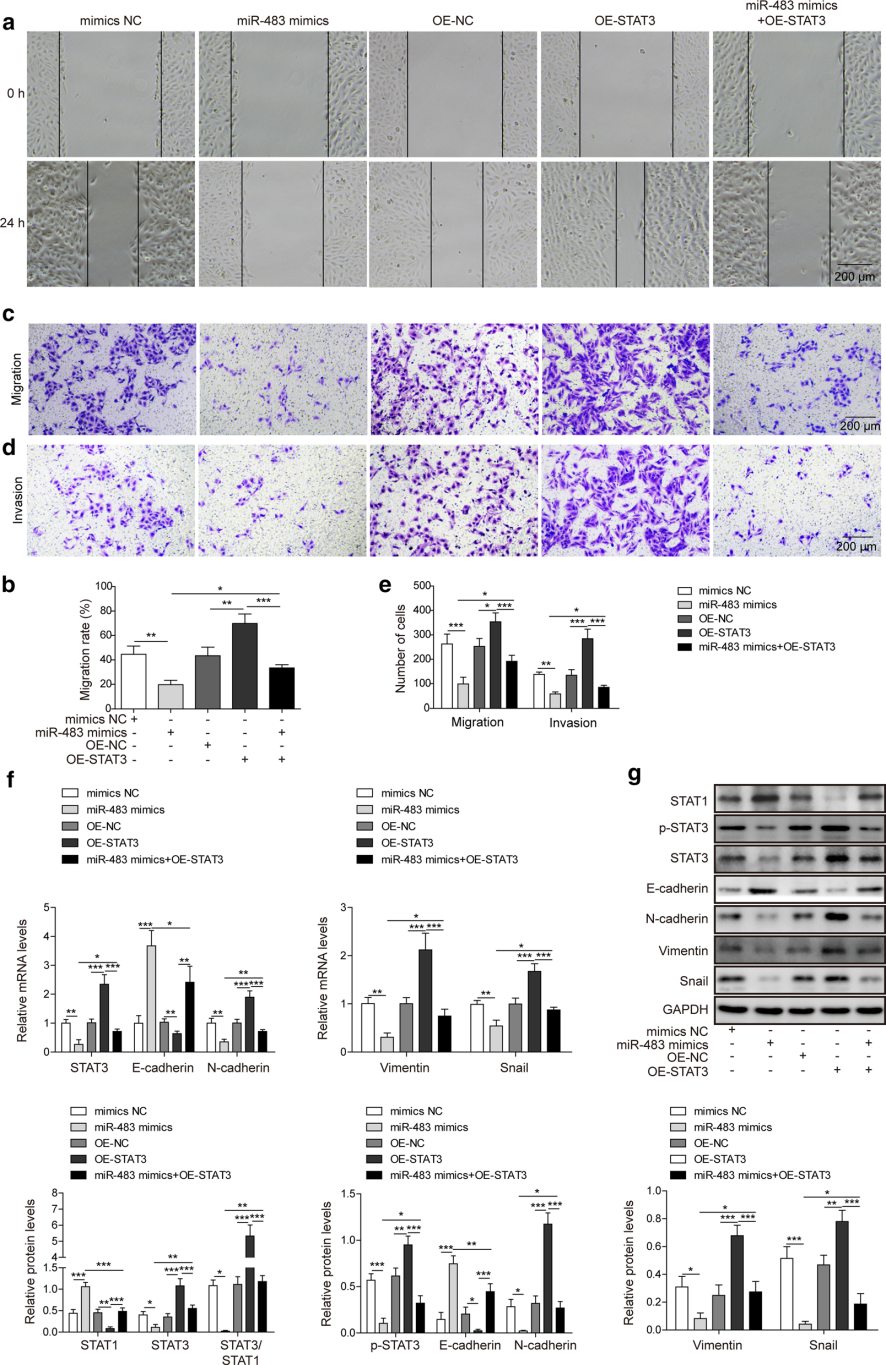
miR-483 inhibits the EMT in osteosarcoma cells by suppressing STAT3 expression.U2OS cells were transfected with mimics NC, miR-483 mimics, empty vector (OE-NC), a STAT3 overexpression plasmid (OE-STAT3), or miR-483 mimics plus the STAT3 overexpression plasmid. **a**–**c**, **e** The migration of U2OS cells was measured using the scratch wound healing experiment (**a**, **b**) or transwell migration experiment (**c**, **e**). Scale bar: 200 μm. **d**, **e** The invasion of U2OS cells was determined using a transwell invasion assay. Scale bar: 200 μm. **f** The relative expression of STAT3, E-cadherin, N-cadherin, Vimentin, and Snail in U2OS cells was determined using qRT-PCR. The mRNA levels were normalized to the GAPDH mRNA. **g** The phosphorylation of STAT3 and the levels of STAT3, STAT1, E-cadherin, N-cadherin, Vimentin, and Snail in U2OS cells were examined using western blotting. GAPDH was employed as the input control. All experiments described in this study were performed at least three times, which referred to biological replicates. Each biological replicate consisted of 3 wells of cells at the same passage, which referred to technical replicates. Error bars denoted mean ± SD. *p* values were determined using one-way analysis of variance (ANOVA) followed by Tukey’s post hoc test. ***p < 0.001, **p < 0.01, and *p < 0.05

We over-expressed STAT3 in U2OS cells (OE-STAT3) through lentivirus-mediated transfection to directly substantiate the finding that miR-483 inhibits the EMT in osteosarcoma cells by suppressing STAT3 expression. Next, wound healing and transwell assays were conducted with the following five types of cells to evaluate the effect of miR-483 and STAT3 overexpression on the migration and invasion of U2OS cells: U2OS cells transfected with the mimics NC, U2OS cells transfected with miR-483 mimics, U2OS cells transfected with the empty vector (OE-NC), U2OS cells transfected with OE-STAT3 and U2OS cells transfected with miR-483 mimics and OE-STAT3. The overexpression of miR-483 significantly impaired the migration of U2OS cells in the wound healing assay (p < 0.01). In contrast, STAT3 overexpression increased the migration of U2OS cells (p < 0.01). Moreover, simultaneous ectopic expression of STAT3 counteracted the effects of miR-483 on cell migration (Fig. 3a, b; p < 0.05). Consistently, the transwell migration assay revealed a similar result for the changes in the migration of U2OS cells induced by miR-483 and STAT3 (Fig. 3c and e; p < 0.001 for miR-483 mimics, p < 0.05 for STAT3 overexpression, p < 0.001 for miR-483 plus STAT3 overexpression in migration). Additionally, the invasion of U2OS cells was also reduced by miR-483 mimics and increased by STAT3 overexpression (Fig. 3d and e; p < 0.01 for miR-483 mimics; p < 0.001 for STAT3 overexpression, miR-483 plus STAT3 overexpression in invasion, respectively). We further analysed the effect of miR-483 and STAT3 overexpression on the EMT using qRT-PCR and western blotting. STAT3 expression was suppressed by miR-483 mimics (p < 0.01 in qRT-PCR; p < 0.05 in western blotting) and restored after STAT3 overexpression (p < 0.001 in qRT-PCR and western blotting). Moreover, N-cadherin, Vimentin and Snail were all downregulated in the presence of miR-483 mimics (p < 0.01 for N-cadherin, Vimentin, Snail in qRT-PCR, respectively; p < 0.05 for N-cadherin and Vimentin in western blotting, respectively; p < 0.001 for Snail in western blotting). Nevertheless, they were all upregulated upon STAT3 overexpression (p < 0.001 for N-cadherin, Vimentin, and Snail in qRT-PCR, respectively; p < 0.001 for N-cadherin and Vimentin in western blotting, respectively; p < 0.05 for Snail in western blotting), and STAT3 overexpression in the presence of miR-483 partially restored their levels (p < 0.001 for N-cadherin, Vimentin, and Snail in qRT-PCR, respectively; p < 0.001 for N-cadherin, Vimentin, and Snail in western blotting, respectively). However, E-cadherin expression was increased by miR-483 mimics (p < 0.001 in qRT-PCR and western blotting) but was partially repressed by STAT3 overexpression (p < 0.01 in qRT-PCR; p < 0.05 in western blotting) (Fig. 3f, g). Notably, STAT3 phosphorylation was also reduced in cells expressing the miR-483 mimics (p < 0.001), nevertheless, it was increased in STAT3-overexpressing cells (Fig. 3g; p < 0.01). As a tumour suppressor, STAT1 enhances the immune response, suppresses proliferation and induces the apoptosis of tumour cells [31], therefore, we also measured its expression. The overexpression of miR-483 significantly increased STAT1 expression (p < 0.001) and decreased the ratio of STAT3/STAT1 (p < 0.05), whereas STAT3 overexpression in the presence of miR-483 restrained these effects (p < 0.001) (Fig. 3g). Thus, miR-483 inhibited the EMT of osteosarcoma cells by restraining STAT3 expression and inducing STAT1 expression.

### The lncRNA NEAT1 sponges miR-483 and suppresses its expression

**Fig. 4:**
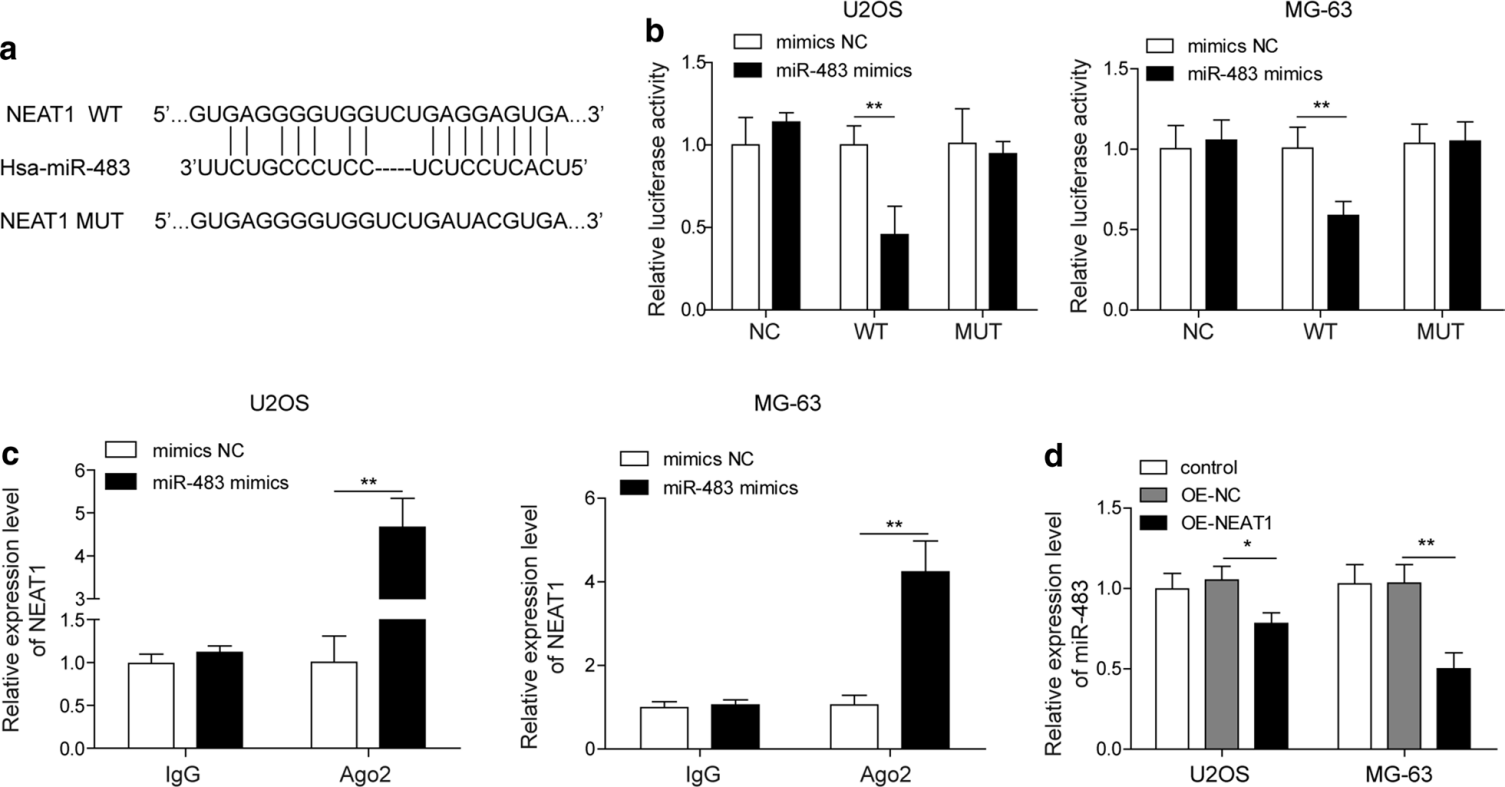
The lncRNA NEAT1 sponges miR-483 and suppresses its expression. **a** The putative binding site for miR-483 in the NEAT1 according to the bioinformatics prediction from the Starbase database. **b** U2OS and MG-63 cells were transfected with a luciferase plasmid containing the wild type or mutant 3′UTR of NEAT1 as along with miR-483 mimics or mimic NC. The interaction between miR-483 and the 3′UTR of NEAT1 was assessed using the dual luciferase assay. **c** U2OS and MG-63 cells were transfected with miR-483 mimics or mimic NC, and the interaction between NEAT1 and miR-483 was confirmed using an RIP assay. **d** U2OS and MG-63 cells were untransfected or transfected with a negative control vector (OE-NC) or NEAT1 overexpression plasmid (OE-NEAT1), and the relative expression of miR-483 was determined using qRT-PCR. The expression of miR-483 was normalized to U6. All experiments described in this study were performed at least three times, which referred to biological replicates. Each biological replicate consisted of 3 wells with cells of the same passage, which referred to technical replicates. Error bars denoted mean ± SD. *p* values were calculated using unpaired two-tailed Student’s *t*-test (**b**, **c**) or one-way analysis of variance (ANOVA) followed by Tukey’s post hoc test (**d**). **p < 0.01 and *p < 0.05

We also used the Starbase database to predict potential targets of NEAT1 and ascertain its role in regulating the EMT of osteosarcoma cells. Surprisingly, miR-483 was one target of NEAT1 (Fig. 4a). We generated luciferase reporter constructs harbouring the wild type of NEAT1 or the mutated NEAT1, which were then transfected into U2OS and MG-63 cells with miR-483 mimics or mimics NC, respectively, to determine the reciprocal regulation between miR-483 and NEAT1. Co-transfection of NEAT1-WT and miR-483 mimics significantly reduced the luciferase activity (p < 0.01 for both U2OS and MG-63 cells), whereas the transfection of the binding site mutation robustly abolished the miR-483-induced suppression of luciferase activity, strongly suggesting that miR-483 was a target of NEAT1 (Fig. 4b). Moreover, we performed an RIP assay to analyse the RNAs that were associated with miR-483. The amount of precipitated NEAT1 was substantially increased in miR-483 mimics-transfected U2OS and MG-63 cells compared with mimics NC-transfected cells (Fig. 4c; p < 0.01 for both U2OS and MG-63 cells). In addition, we overexpressed NEAT1 in U2OS and MG-63 cells via lentivirus-mediated transfection, and the qRT-PCR experiment revealed that NEAT1 overexpression downregulated miR-483 expression compared with the control group of cells (Fig. 4d; p < 0.05 for U2OS cells; p < 0.01 for MG-63 cells). Based on these data, the lncRNA NEAT1 sponged miR-483 and suppressed its expression.

### The lncRNA NEAT1 promotes the EMT in osteosarcoma cells by inhibiting miR-483

**Fig. 5:**
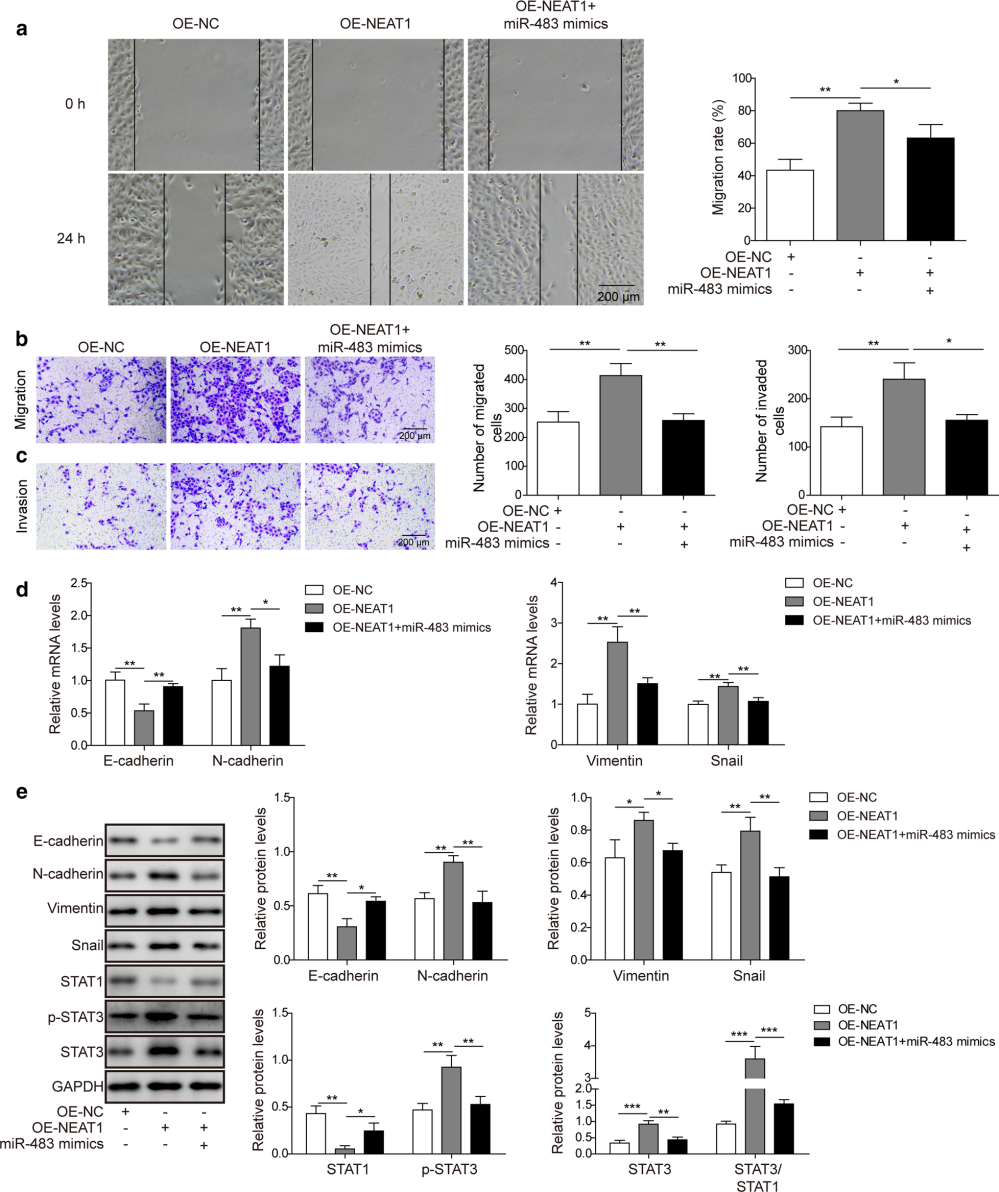
The lncRNA NEAT1 promotes the EMT in osteosarcoma cells by inhibiting miR-483.U2OS cells were transfected with a negative control plasmid (OE-NC), NEAT1 overexpression plasmid (OE-NEAT1) or NEAT1 overexpression plasmid plus miR-483 mimics. **a**, **b** The migration of transfected U2OS cells was measured using a scratch wound healing experiment (**a**) or transwell migration experiment (**b**). Scale bar: 200 μm. **c** The invasion of transfected U2OS cells was determined using transwell invasion assays. Scale bar: 200 μm. **d** The relative expression levels of N-cadherin, E-cadherin, Vimentin, and Snail in transfected U2OS cells were determined using qRT-PCR and normalized to the GAPDH mRNA. **e** The levels of N-cadherin, E-cadherin, Vimentin, Snail, STAT1, STAT3 and the phosphorylation of STAT3 in transfected U2OS cells were examined using western blotting. GAPDH was employed as the input control. All experiments described in this study were performed at least three times, which referred to biological replicates. Each biological replicate consisted of 3 wells with cells of the same passage, which referred to technical replicates. Error bars denoted mean ± SD. *p* values were calculated using one-way analysis of variance (ANOVA) followed by Tukey’s post hoc test. ***p < 0.001, **p < 0.01 and *p < 0.05

In the experiments described above, miR-483 inhibited the EMT of osteosarcoma cells. Since miR-483 was a target of NEAT1, we speculated that NEAT1 might promote the EMT of osteosarcoma cells. We overexpressed NEAT1 in U2OS cells in the absence or presence of miR-483 mimics, and then conducted the wound healing and transwell experiments to examine the effect of NEAT1 overexpression on the migration and invasion of osteosarcoma cells and to verify this hypothesis. NEAT1 overexpression evidently increased the migration of U2OS cells compared with control cells in the wound healing assay (p < 0.01). However, this increased migration was diminished following the additional overexpression of miR-483 (Fig. 5a; p < 0.05). Consistent with these findings, the changes in cell migration were confirmed by the results of transwell migration assay (Fig. 5b; p < 0.01 for OE-NEAT1 and OE-NEAT1 plus miR-483 mimics). Additionally, the transwell invasion assay revealed increased invasion of U2OS cells overexpressing NEAT1, which was subsequently repressed by the co-transfection of miR-483 mimics (Fig. 5c; p < 0.01 for OE-NEAT1; p < 0.05 for OE-NEAT1 plus miR-483 mimics). Meanwhile, we further assessed the relative expression levels of EMT-related genes using qRT-PCR, and NEAT1 overexpression upregulated the expression of N-cadherin, Vimentin and Snail and downregulated the expression of E-cadherin (p < 0.01 for N-cadherin, Vimentin, Snail and E-cadherin, respectively), indicating that the EMT was induced in these cells. Nevertheless, simultaneous overexpression of NEAT1 and miR-483 counteracted the effect of NEAT1 and restrained the EMT process (Fig. 5d; p < 0.01 for E-cadherin, Vimentin, Snail; p < 0.05 for N-cadherin). Finally, the results of western blotting experiments coincided with the qRT-PCR results and further confirmed the functions of NEAT1 and miR-483 in regulating the EMT in osteosarcoma cells (p < 0.01 for E-cadherin, N-cadherin, Snail in OE-NEAT1 group; p < 0.05 for Vimentin in OE-NEAT1 group; p < 0.01 for N-cadherin and Snail in OE-NEAT1 plus miR-483 mimics group; p < 0.05 for E-cadherin and Vimentin in OE-NEAT1 plus miR-483 mimics group). Moreover, STAT1 expression was reduced upon the inhibition of miR-483 by NEAT1 (p < 0.01), whereas the level and phosphorylation of STAT3 were both increased upon NEAT1 overexpression (p < 0.01 for p-STAT3; p < 0.001 for STAT3). However, the overexpression of miR-483 mimics increased STAT1 levels (p < 0.05) and reduced the level and phosphorylation of STAT3 (p < 0.01 for STAT3 and p-STAT3) (Fig. 5e). Overall, this evidence substantiated the hypothesis that the lncRNA NEAT1 promotes the EMT of osteosarcoma cells by sponging miR-483.

### The lncRNA NEAT1 upregulates STAT3 to promote the EMT in osteosarcoma cells by inhibiting miR-483

**Fig. 6:**
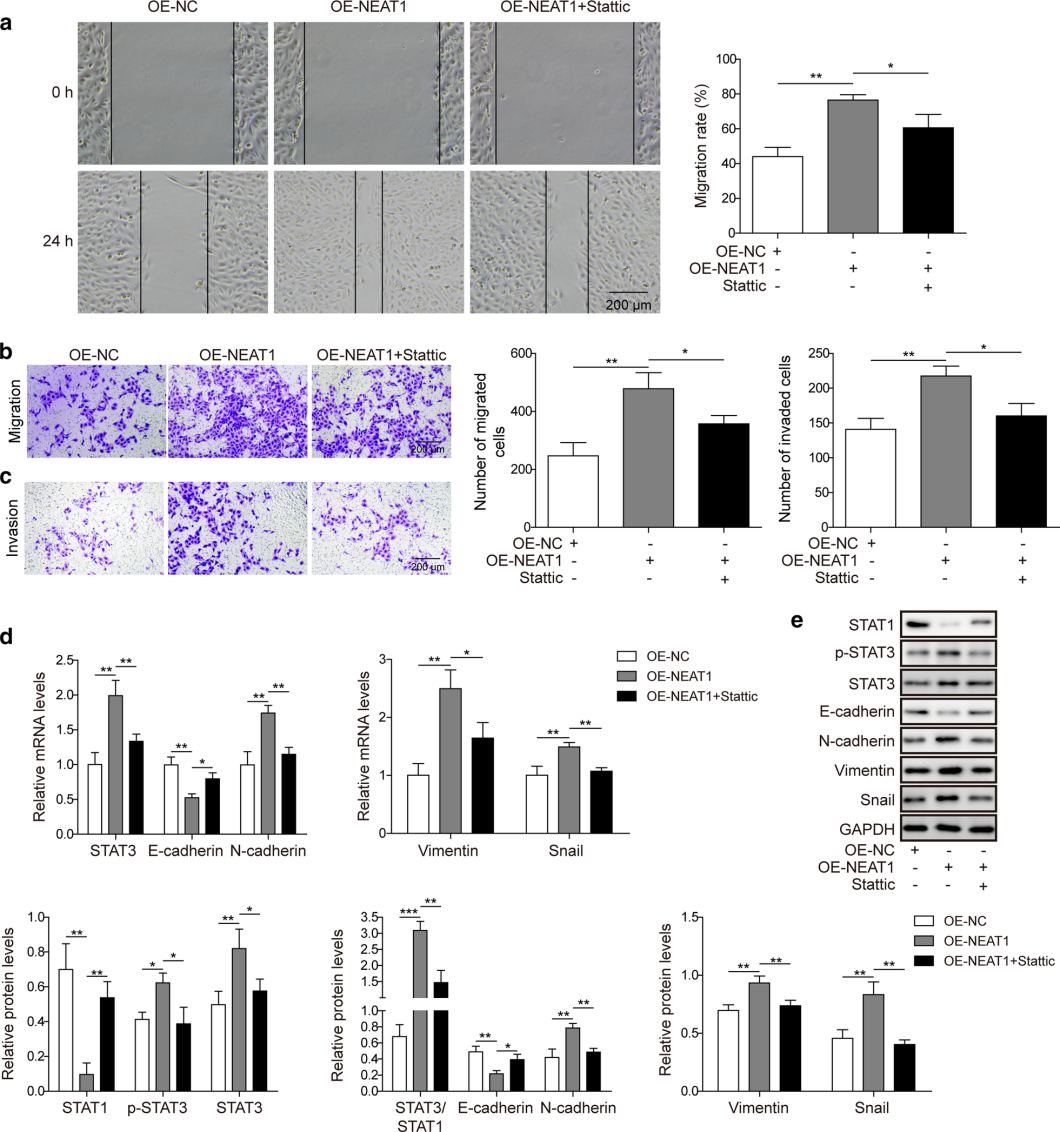
The lncRNA NEAT1 upregulates STAT3 to promote the EMT in osteosarcoma cells by inhibiting miR-483. U2OS cells were transfected with a negative control (OE-NC) or NEAT1 overexpression plasmid (OE-NEAT1). NEAT1-overexpressing U2OS cells were then cultured in the absence or presence of 15 µM Stattic (STAT3 inhibitor). **a**, **b** The migration of U2OS cells receiving different treatments was examined using a scratch wound healing experiment (**a**) or transwell migration experiment (**b**). Scale bar: 200 μm. **c** The invasion of U2OS cells subjected to the indicated treatments was assessed using the transwell invasion experiment. Scale bar: 200 μm. **d** The relative expression of STAT3, N-cadherin, E-cadherin, Vimentin, and Snail in U2OS cells was determined using qRT-PCR and normalized to the GAPDH mRNA. **e** The phosphorylation of STAT3 and the levels of STAT3, STAT1, N-cadherin, E-cadherin, Vimentin, and Snail in U2OS cells were examined using western blotting. GAPDH was employed as the input control. All experiments described in this study were performed at least three times, which referred to biological replicates. Each biological replicate consisted of 3 wells with cells of the same passage, which referred to technical replicates. Error bars denoted mean ± SD. *p* values were calculated using one-way analysis of variance (ANOVA) followed by Tukey’s post hoc test. ***p < 0.001, **p < 0.01, and *p < 0.05

After a careful analysis of the evidence described above, we hypothesized that NEAT1 upregulated STAT3 by inhibiting miR-483 expression, thus promoting the EMT of osteosarcoma cells. We treated NEAT1-overexpressing U2OS cells with 15 µM Stattic, a STAT3 inhibitor, to verify our hypothesis. As described above, NEAT1 overexpression increased the migration and invasion of U2OS cells (p < 0.01). However, the inhibition of STAT3 activity by Stattic potently blocked the migration of U2OS cells, even in the presence of excess NEAT1, as evidenced by the results of the wound healing experiment (Fig. 6a; p < 0.05) and transwell migration experiment (Fig. 6b; p < 0.05). Additionally, the Stattic treatment significantly impaired the invasion of U2OS cells, despite the effects of the NEAT1 on promoting cell invasion (Fig. 6c; p < 0.05). Next, we analysed the expression of EMT markers in U2OS cells treated with Stattic using qRT-PCR and western blotting. The inhibition of STAT3 activity with Stattic reduced STAT3 expression (p < 0.01 for qRT-PCR; p < 0.05 for western blotting) and phosphorylation (p < 0.05), reduced the expression N-cadherin, Vimentin and Snail (p < 0.01 for N-cadherin and Snail, p < 0.05 for Vimentin in qRT-PCR; p < 0.01 for N-cadherin, Vimentin, and Snail in western blotting), and increased the expression of E-cadherin and STAT1 (p < 0.05 for E-cadherin in qRT-PCR; p < 0.05 for E-cadherin, p < 0.01 for STAT1 in western blotting, respectively) (Fig. 6d, e). In summary, these results confirmed that NEAT1 promoted the EMT of osteosarcoma cells by upregulating STAT3 expression and increasing its phosphorylation, which was mediated by the inhibition of miR-483 expression.

### The lncRNA NEAT1/miR-483/STAT3 axis contributes to the liver and lung metastases of osteosarcoma in mice

**Fig. 7:**
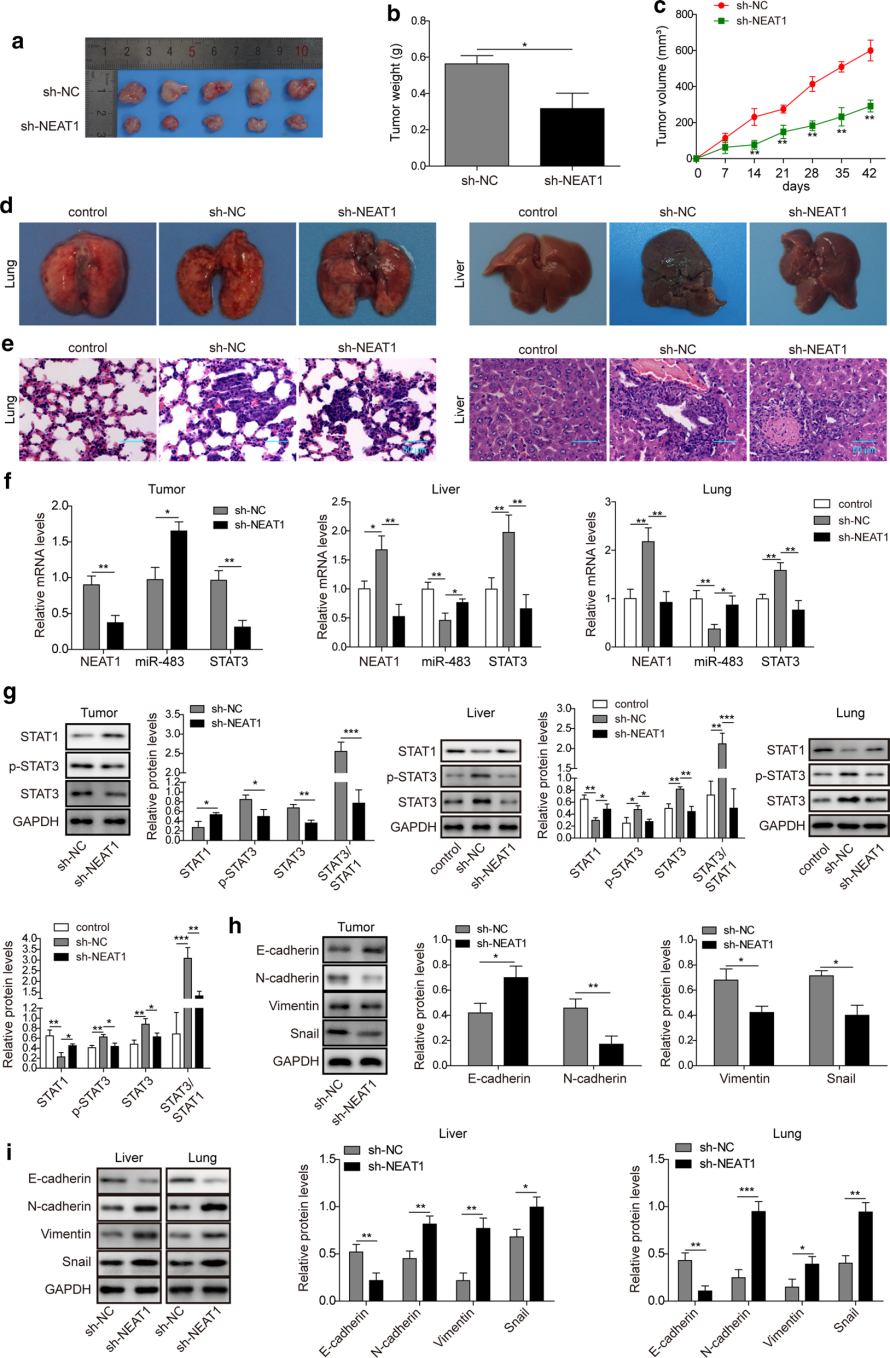
The lncRNA NEAT1/miR-483/STAT3 axis contributes to the liver and lung metastases of osteosarcoma in mice. U2OS cells were transfected with the sh-NC or sh-NEAT1 plasmid. Subsequently, cells were subcutaneously injected into nude mice to establish liver and lung metastases of osteosarcoma (n = 5). Untreated nude mice served as the negative control. **a** Representative images of resected tumours from tumour-bearing mice. **b** The weight of resected tumours from tumour-bearing mice. **c** The tumour volume of tumours was monitored every 7 days for 6 weeks. **d** Representative images showing the distribution, quantity, and volume of metastatic osteosarcoma tumours in the lung (left panel) and liver (right panel) from different groups of mice, normal lung and liver tissues in mice without any treatment were served as the control. **e** H&E staining showing the invasion of metastatic osteosarcoma tumours, normal lung and liver tissues in mice without any treatment were served as the control. Scale bar: 50 μm. **f** The relative expression of NEAT1, miR-483 and STAT3 in primary tumour tissues and metastatic tumour tissues was examined using qRT-PCR. The NEAT1 and STAT3 mRNAs were normalized to the GAPDH mRNA, miR-483 was normalized to U6. Normal lung and liver tissues in mice without any treatment were served as the control in liver and lung panel. **g** The levels of STAT3 and STAT1 and the phosphorylation of STAT3 in primary tumour tissues and metastatic tumour tissues were assessed using western blotting. GAPDH was employed as the input control. Normal lung and liver tissues in mice without any treatment were served as the control in liver and lung panel. **h** The levels of N-cadherin, E-cadherin, Vimentin, and Snail in primary tumour tissues were assessed using western blotting. GAPDH was employed as the input control. **i** The levels of N-cadherin, E-cadherin, Vimentin, and Snail in metastatic tumour tissues in the lung and liver were assessed using western blotting. GAPDH was employed as the input control. All experiments described in this study were performed at least three times, which referred to biological replicates. Each biological replicate consisted of 3 wells with cells of the same passage, which referred to technical replicates. Error bars denoted mean ± SD. *p* values were calculated using unpaired two-tailed Student’s *t*-test or one-way analysis of variance (ANOVA) followed by Tukey’s post hoc test. ***p < 0.001, **p < 0.01, and *p < 0.05

Next, we generated stable NEAT1 KD U2OS cells by transducing them with a lentivirus carrying the sh-NEAT1 to further confirm our hypothesis in vivo. Afterwards, control shRNA (sh-NC)-transfected or NEAT1 KD U2OS cells were subcutaneously injected into the left outer flank of BALB/c nude mice to establish liver and lung metastases of osteosarcoma. Six weeks later, the mice were sacrificed to examine the growth of osteosarcoma at the injection site and the metastases in the liver and lung. Mice injected with sh-NEAT1-transfected U2OS cells had significantly smaller primary tumour volumes and weights than mice injected with sh-NC U2OS cells (Fig. 7a, b; p < 0.05). The primary tumour volume progression data revealed a slower growth rate of osteosarcoma derived from sh-NEAT1-transfected U2OS cells than tumours derived from sh-NC-transfected U2OS cells (Fig. 7c; p < 0.01 at day 14, 21, 28, 35, 42, respectively). A small number of metastatic nodules were observed in the lung and liver of mice injected with sh-NEAT1-transfected U2OS cells (Fig. 7d). Consistent with these findings, HE staining revealed a smaller extent of tumour cell invasion in the lung and liver of nude mice inoculated with sh-NEAT1-transfected U2OS cells (Fig. 7e). Moreover, we performed qRT-PCR and western blotting of primary (tumour) and metastatic tumour (lung and liver) samples, as well as normal tissues (normal lung and liver tissues in negative control mice without any treatment). The levels of NEAT1, STAT3 and the phosphorylation of STAT3 were evidently decreased (p < 0.01 for NEAT1 in primary tumor and metastatic tumor in lung and liver by qRT-PCR; p < 0.01 for STAT3 in primary tumor and metastatic tumor in lung and liver by qRT-PCR; p < 0.01 for STAT3 in primary tumor and metastatic tumor in liver in western blotting; p < 0.05 for STAT3 in metastatic tumor in lung in western blotting; p < 0.05 for p-STAT3 in primary tumor and metastatic tumor in lung and liver in western blotting), while the levels of miR-483 and STAT1 were increased in primary tumours derived from sh-NEAT1-transfected U2OS cells compared with sh-NC-transfected U2OS cells (p < 0.05 for miR-483 in qRT-PCR; p < 0.05 for STAT1 in western blotting) (Fig. 7f, g, Tumour Panel). What’s more, the levels and phosphorylation of STAT3 and NEAT1 expression were increased (p < 0.05 and p < 0.01 for NEAT1 expression in liver and lung metastatic tumors by qRT-PCR, respectively; p < 0.01 for STAT3 expression in liver and lung metastatic tumours by qRT-PCR, respectively; p < 0.01 for STAT3 expression in liver and lung metastatic tumours by western blotting, respectively; p < 0.05 and p < 0.01 for p-STAT3 in liver and lung metastatic tumours by western blotting, respectively), but the levels of miR-483 and STAT1 were decreased in metastatic tumours (liver and lung) derived from sh-NC-transfected U2OS cells compared with normal lung and liver tissues in negative control mice without any treatment (p < 0.01 for miR-483 expression in liver and lung metastatic tumours by qRT-PCR, respectively; p < 0.01 for STAT1 expression in liver and lung metastatic tumours by western blotting, respectively). In contrast, NEAT1 KD restrained the above changes in metastatic tumours (liver and lung) (Fig. 7f, g, liver and lung panel). In addition, western blotting results revealed decreased levels of EMT-associated cell surface markers and transcription factors in primary tumours derived from sh-NEAT1-transfected U2OS cells, but the levels of epithelial markers were increased (Fig. 7h; p < 0.01 for N-cadherin, p < 0.05 for E-cadherin, Vimentin, and Snail, respectively). In the metastatic tumours (liver and lung) derived from sh-NEAT1-transfected U2OS cells, the expression of the epithelial marker E-cadherin was downregulated, while the expression of N-cadherin, Vimentin and Snail was upregulated, indicating that the MET process was suppressed in the metastatic sites (Fig. 7i; p < 0.01, p < 0.01, p < 0.01, and p < 0.05 for E-cadherin, N-cadherin, Vimentin, and Snail in liver, respectively; p < 0.01, p < 0.001, p < 0.05, and p < 0.01 for E-cadherin, N-cadherin, Vimentin, and Snail in liver, respectively). Taken together, NEAT1 promoted the liver and lung metastases of osteosarcoma in vivo through the miR-483/STAT3 axis.

## Discussion

The EMT is a crucial process contributing to the metastasis and invasion of malignant tumours [32–34], including osteosarcoma. However, the regulation of EMT in osteosarcoma has been unclear. Recently, numerous studies have substantiated that both STAT3 and the EMT are closely related to the initiation and development of malignant tumours [35–37], and the STAT3 protein has the potential to become an ideal drug target for anti-tumour therapy. Meanwhile, STAT3 facilitates the proliferation and metastasis of osteosarcoma [7, 38], and the inhibition of STAT3 activity with various compounds or natural products suppresses the proliferation and migration of osteosarcoma cells or induces cell cycle arrest, apoptosis and autophagy [39, 40]. In the present study, overexpression of STAT3 enhanced the migration and invasion of osteosarcoma cells by increasing the expression of N-cadherin, Vimentin, Snail and reducing the expression of E-cadherin to subsequently promote the EMT process. The results from our study were consistent with previous findings. However, overexpression of miR-483 could downregulate STAT3 expression, thereby inhibiting the metastasis and invasion of osteosarcoma cells. More importantly, miR-483 expression was downregulated in osteosarcoma cell lines and osteosarcoma tumour tissues in the present study. We performed bioinformatics analyses using the Starbase database to further investigate the association between miR-483 and STAT3 and identified that the 3′UTR of STAT3 mRNA contained a binding site for miR-483, suggesting that STAT3 was negatively modulated by miR-483. The association between STAT3 and miR-483 was verified by a dual luciferase assay, and the reversed expression pattern between them further supported the finding that miR-483 targeted STAT3 and reduced its expression. Moreover, exogenous expression of STAT3 partially blocked the miR-483-mediated suppression of the EMT in osteosarcoma cells, confirming that STAT3 functioned in the downstream of miR-483. Based on accumulating evidence, STAT3 activation by phosphorylation promotes the metastatic progression of various solid tumours [41–43]. Interestingly, the phosphorylation of STAT3 was decreased in miR-483-overexpressing cells, indicating that STAT3 activation was negatively regulated by miR-483 and probably mediated by the reduced expression of the STAT3 protein due to miR-483 overexpression. As one homologue of STAT3, STAT1 usually functions as a tumour suppressor. In our study, STAT1 expression was increased following miR-483 overexpression, whereas its expression was repressed after STAT3 overexpression. Thus, a low STAT3/STAT1 ratio indicated the impaired migration, invasion and EMT of osteosarcoma cells, consistent with a previous report describing the correlation between STAT3/STAT1 ratio and patient survival [44]. Obviously, miR-483 inhibited the EMT of osteosarcoma by targeting STAT3. Also, we are the first to study the interaction between miR-483 and STAT3.

In recent years, the dysregulation of lncRNAs has been identified in various tumour tissues. As one newly discovered lncRNA, NEAT1 also participates in the occurrence and development of various tumours and regulates tumour cell invasion and metastasis [25, 26, 45]. Notably, lncRNAs exert their functions by regulating the expression of target genes at epigenetic, transcriptional, and posttranscriptional levels [46, 47]. In the present study, NEAT1, although it did not directly bind to STAT3, still contained a putative binding site for miR-483, similar to STAT3. Using dual luciferase assay and RIP experiments, we identified miR-483 as a target of NEAT1, and the exogenous expression of NEAT1 reduced miR-483 expression, subsequently upregulating the expression of N-cadherin, Vimentin and Snail and promoting the EMT in osteosarcoma cells. Our results further confirmed the sponging of miR-483 by NEAT1, which has also been reported by Xia Sang in ovarian granulosa cells [48]. Using this mechanism, NEAT1 finely restrains the expression of miR-483 and promotes the proliferation and migration of osteosarcoma cells. We also performed western blotting experiments using NEAT1-overexpressing U2OS cells to assess the effect of NEAT1 on STAT3 and STAT1 expression. STAT1 expression was indeed suppressed upon the inhibition of miR-437 by overexpressed NEAT1, whereas the levels of total and phosphorylated STAT3 were both increased upon NEAT1 overexpression. Moreover, miR-483 mimics plus NEAT1 overexpression counteracted the effect of NEAT1 on the expression of STAT1 and STAT3 and the phosphorylation of STAT3, further confirming that the effect of NEAT1 on STAT1 and STAT3 expression was mediated by miR-483. In summary, these three components constituted a network that regulates the EMT of osteosarcoma cells. Although our study is not the first study reporting the association between NEAT1 and miR-483 [48], it describes the regulation of STAT3 by miR-483 and their functions in regulating the EMT and metastasis of osteosarcoma cells for the first time. Our findings are also consistent with previous reports that overexpression of NEAT1 increases the proliferation, invasion and metastasis of oesophageal squamous cell carcinoma [49], while NEAT1 knockdown impairs cell invasion and migration and reduces the levels of EMT-associated proteins in renal cell carcinoma [50].

The NEAT1/miR-483/STAT3 axis identified in the present study played a crucial role in modulating the EMT in osteosarcoma cells, and a similar axis with different miRNAs as the mediator has been shown to participate in the progression of various tumours. For instance, Yamei Pang found that the NEAT1/miR-124/STAT3 axis regulated the progression of breast cancer, and NEAT1 and STAT3 displayed similar functions as in our study [51]. According to Peixin Dong, NEAT1 facilitated the progression of aggressive endometrial cancer by repressing miR-361 expression and increasing STAT3 signalling [52]. In the study by Shuai Wang, the NEAT1 paraspeckle promoted the progression of human hepatocellular carcinoma by increasing IL-6/STAT3 signalling [53]. Moreover, STAT3 may function as one positive feedback regulator that induces the upregulation of NEAT1 as a competing endogenous RNAs (ceRNA) that then facilitates abdominal aortic aneurysm formation by targeting the miR-4688/TULP3 axis [20]. The forward signalling cascades and reverse feedback loop between NEAT1 and STAT3 have been verified in different tumours, and some tissue-specific or universal miRNAs will fill in the gap and form one tissue/tumour-specific NEAT1/miRNA/STAT3 axis for the indicated tumour.

We further established the liver and lung metastases model of osteosarcoma in nude mice to verify the in vitro data. The primary tumour weight and volume data indicated that NEAT1 KD significantly suppressed tumour growth. Furthermore, the number and volume of metastatic osteosarcoma lesions in nude mice injected with NEAT1 KD cells were significantly lower than in mice injected with control shRNA-transfected cells, suggesting that NEAT1 sponged miR-483 and then upregulated STAT3 expression to promote the proliferation, migration and invasion of osteosarcoma cells. Another interesting finding was the involvement of the NEAT1/miR-183/STAT3 axis in the MET process, which is the opposite process of the EMT and was proposed as a crucial mechanism underlying the formation of metastatic tumours [54–57]. Previous studies have mainly focused on the mechanism regulating the NEAT1/miRNA/STAT3 axis during the EMT [58]. However, our study also assessed the function of the NEAT1/miR-183/STAT3 axis in the MET process in metastatic tumours. NEAT1 was required for both the EMT and the MET. NEAT1 KD significantly impaired both the EMT and MET processes in primary tumours and metastatic tumours respectively during the metastasis of osteosarcoma, indicating versatile roles for NEAT1. In addition, our study revealed a similar function of NEAT1 in the promotion of the EMT as reported by other groups that mainly examined breast cancer cells and lung cancer cells [56, 57, 59, 60]. Nevertheless, osteosarcoma is a type of sarcoma derived from mesenchymal tissues, which usually displays the metastable phenotype [61]. This intermediate state enables these cells to become either more mesenchymal or epithelial under specific conditions, which further enhances the survival, metastasis, and aggressiveness of sarcomas. We speculated that the microenvironment in the liver and lung promoted the MET process instead of the EMT, as indicated by the increase in E-cadherin expression and decrease in the expression of N-cadherin, Vimentin, and Snail. Based on the preliminary data, NEAT1 was required for this conversion, since NEAT1 KD repressed the MET process. However, we did not analyse the detailed mechanism by which NEAT1 regulates the MET in the present study, and this interesting question might be investigated by our lab in a subsequent study. Based on our data, strategies targeting NEAT1 with small molecule inhibitors or siRNAs will potentially interfere with both the MET and EMT processes during the metastasis of osteosarcoma.

Also, most of these conclusions were drawn using U2OS cells due to limited resources and time available in our lab and should be confirmed using another osteosarcoma cell line, although both the in vitro and in vivo data fully supported our conclusions. In the future, we will perform studies with two osteosarcoma cell lines to reinforce our results.

## Conclusions

The lncRNA NEAT1/miR-483/STAT3 axis regulated the EMT and metastasis of osteosarcoma cells, which might represent new drug targets for the treatment of osteosarcoma in the future. However, further studies are needed to elucidate whether NEAT1 and miR-483 participate in the mechanism regulating the biological behaviour of osteosarcoma cells through other signalling pathways, which will be further explored in our future studies.

## Data Availability

All data generated or analyzed during this study are included in this published article.

## Abbreviations

LncRNAs: Long non-coding RNAs
miRNAs: MicroRNAs
EMT: Epithelial-mesenchymal transition
STAT3: Signal transducer and activator of transcription-3
STAT1: Signal transducer and activator of transcription-1
RIP: RNA immunoprecipitation
NEAT1: Nuclear paraspeckle assembly transcript 1
NSCLC: Non-small cell lung cancer
MSC: Mesenchymal stem cells
CRC: Colorectal cancer cells
ATCC: American Type Cell Culture
FBS: Foetal bovine serum
DMEM: Dulbecco’s Modified Eagle’s Medium
qRT-PCR: Quantitative reverse transcription-polymerase chain reaction
CST Cell: Signaling Technology
PFA: Paraformaldehyde
Ago2: Argonaute2
MET: Mesenchymal- epithelial transition
AEC: Animal Ethics Committee
HE: Haematoxylin & eosin
SD: Standard deviation
ANOVA: Analysis of variance
3′UTR: 3′ untranslated region
